# *In silico* identification of bacteriocin gene clusters in the gastrointestinal tract, based on the Human Microbiome Project’s reference genome database

**DOI:** 10.1186/s12866-015-0515-4

**Published:** 2015-09-16

**Authors:** Calum J. Walsh, Caitriona M. Guinane, Colin Hill, R. Paul Ross, Paul W. O’Toole, Paul D. Cotter

**Affiliations:** Teagasc Food Research Centre, Moorepark, Fermoy Cork, Ireland; APC Microbiome Institute, University College Cork, Cork, Ireland; School of Microbiology, University College Cork, Cork, Ireland

## Abstract

**Background:**

The human gut microbiota comprises approximately 100 trillion microbial cells which significantly impact many aspects of human physiology - including metabolism, nutrient absorption and immune function. Disturbances in this population have been implicated in many conditions and diseases, including obesity, type-2 diabetes and inflammatory bowel disease. This suggests that targeted manipulation or shaping of the gut microbiota, by bacteriocins and other antimicrobials, has potential as a therapeutic tool for the prevention or treatment of these conditions. With this in mind, several studies have used traditional culture-dependent approaches to successfully identify bacteriocin-producers from the mammalian gut. *In silico*-based approaches to identify novel gene clusters are now also being utilised to take advantage of the vast amount of data currently being generated by next generation sequencing technologies. In this study, we employed an *in silico* screening approach to mine potential bacteriocin clusters in genome-sequenced isolates from the gastrointestinal tract (GIT). More specifically, the bacteriocin genome-mining tool BAGEL3 was used to identify potential bacteriocin producers in the genomes of the GIT subset of the Human Microbiome Project’s reference genome database. Each of the identified gene clusters were manually annotated and potential bacteriocin-associated genes were evaluated.

**Results:**

We identified 74 clusters of note from 59 unique members of the Firmicutes, Bacteroidetes, Actinobacteria, Fusobacteria and Synergistetes. The most commonly identified class of bacteriocin was the >10 kDa class, formerly known as bacteriolysins, followed by lantibiotics and sactipeptides.

**Conclusions:**

Multiple bacteriocin gene clusters were identified in a dataset representative of the human gut microbiota. Interestingly, many of these were associated with species and genera which are not typically associated with bacteriocin production.

**Electronic supplementary material:**

The online version of this article (doi:10.1186/s12866-015-0515-4) contains supplementary material, which is available to authorized users.

## Background

Bacteriocins are ribosomally synthesized antimicrobial peptides produced by bacteria that are active against other bacteria, either within the same species (narrow spectrum) or across genera (broad spectrum), and to which the producing organism is immune by a specific immunity protein(s) [1]. Some bacteriocins, most notably nisin, have a long history of use as preservatives in the food industry [2] and these antimicrobials are also receiving increased attention as potential alternatives to antibiotics [3].

The intestinal microbiota comprises a dynamic community with 100–1000 phylotypes [4, 5] playing an integral role in gastrointestinal (GI) health and disease [6, 7]. As a consequence of advances in DNA sequencing technologies, there is now a clearer understanding of the composition of the GI microbiota and of associations between specific taxa with health and disease [6, 8]. This knowledge can potentially be utilised through the modulation of the gut microbiota to address certain GI disorders [9, 10]. Bacteriocins are ideal candidates with respect to the targeting of undesirable populations due to their generally low toxicity, high potency and, particularly in the case of gut-associated isolates, the possibility of *in situ* production [11]. There have been some notable proof of concept studies, such as the use of a representative of the sactibiotic group of bacteriocins, thuricin CD, to specifically inhibit *Clostridium difficile* in a distal colon model, without significantly impacting on other members of the microbiota [12]. Similarly, bacteriocin production by the probiotic *Lactobacillus salivarius* UCC118 was shown to be directly responsible for significantly protecting mice against *Listeria monocytogenes* infection [13]. Bacteriocin production has also been investigated to assess the extent to which it can control weight gain as a consequence of changing the composition of the gut microbiota [14, 15].

There are a variety of strategies by which novel bacteriocin producers can be identified [16]. These can be broadly divided into traditional, culture-based approaches and newer, *in silico-*based, strategies. The latter take advantage of the vast amount of data generated by genome and metagenome sequencing projects and the fact that many features of bacteriocin gene clusters, and especially bacteriocin modification genes, are highly conserved. These modification genes encode enzymes responsible for the post-translational modification of Class 1 bacteriocins into their active forms. Other features common to bacteriocin gene clusters include specific immunity genes, ABC transporters for bacteriocin export, and leader cleavage peptidases for removing the leader sequence from the structural prepeptide (for a review see Arnison *et al*. [17]). To date, *in silico* bacteriocin screening strategies have led to the identification of many novel lantibiotic [16, 18–21], microcin [22] and sactibiotic [23] gene clusters of interest. While in a number of instances standard BLAST-based approaches have been employed to identify such clusters, the BAGEL web-based bacteriocin mining tool (http://bagel.molgenrug.nl/) has been a particularly valuable resource [24]. BAGEL combines direct mining for the structural gene with indirect mining for bacteriocin-associated genes. The latter is particularly useful for identifying peptides which undergo significant post-translational modification such as those observed in lantibiotics. The most recent iteration of this tool, BAGEL3 [24], was recently used to evaluate the density and diversity of bacteriocins in the human microbiome [25]. A previous version of this software was, for example, used in the identification of the novel, two-peptide lantibiotic lichenicidin [18] and 24 putative novel lantibiotics from genomic data [20]. BAGEL3 classifies clusters in a manner consistent with the generally accepted approach of dividing bacteriocins on the basis of whether they are modified (class I) or unmodified/minimally modified (class II) [1, 11]. The former can be sub-divided into a number of subclasses including the lantibiotics, sactibiotics, some microcins, bottromycins, and linear azol(in)e-containing peptides (LAPs) [11, 17]. In addition, it also identifies antimicrobial proteins larger than 10 kDa in size (i.e. bacteriolysins, previously referred to as Class III).

Among the large databases of microbiota data that can be screened using *in silico* approaches are those generated by the Human Microbiome Project (HMP). The HMP was established with the goals of characterising the human microbiome, elucidating its role in health and disease, and developing new tools and databases to aid researchers. Among the data generated by the HMP is a reference genome database, which is a collection of genome-sequences from species/strains isolated from a variety of human body sites (http://www.hmpdacc.org/). The gastrointestinal tract (GIT) subset of this reference genome database was chosen as the focus of this study, which aimed to find bacteriocin-producers with the potential to alter the composition of the gut microbiota *in situ*. Indeed, previous culture-based approaches have shown the human gut is a rich reservoir of bacteriocin-producers [26–28]. Here we employ the bacteriocin genome-mining tool BAGEL3 to screen the GIT subset of the HMP reference genome database and identify 74 putative bacteriocin-encoding gene clusters (PBGCs) from 59 unique producers.

## Results and Discussion

### *In silico* screen for putative bacteriocin-encoding gene clusters

**Fig. 1:**
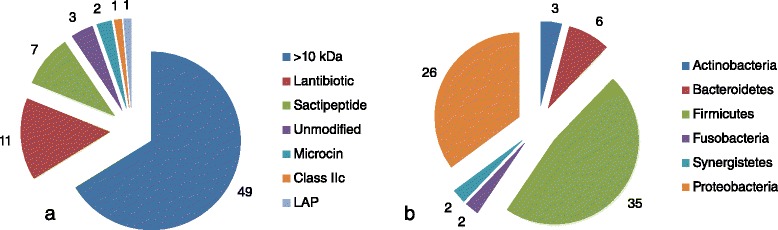
**a** Frequency of bacteriocin class and (**b**) producing phylum among the 74 PBGCs identified

The GIT subset of the HMP reference genome database contained 382 fully sequenced genomes. The bacteriocin mining software tool BAGEL3 initially identified 217 areas of interest (AOIs) from 130 unique putative producers (Additional file 1: Table S1). Subsequent manual annotation and Blast analysis determined that 74 of these were PBGCs (Table 1). The remaining AOIs were eliminated following manual annotation due to the absence of key bacteriocin associated genes. However, we accept the possibility that these gene products may work in concert with other novel bacteriocin-related genes encoded elsewhere on the genome. Selection of the 74 PBGCs was achieved based on the presence of bacteriocin-associated genes, arrangement of those genes in the AOI, and by overall similarity to previously described gene clusters. An overall breakdown of the 74 PBGCs according to phylum and predicted bacteriocin type can be seen in Fig. 1a, b, respectively. The vast majority of PBGCs belonged to members of the Firmicutes and Proteobacteria phyla, and, in the latter case, *Escherichia coli* strains in particular. PBGCs were also identified in the Bacteroidetes, Actinobacteria, Fusobacteria and Synergistetes phyla. The most commonly identified clusters were > 10 kDa bacteriolysins followed by lantibiotics and sactipeptides (Fig. 1).

**Table 1:** Additional information on PBGCs and whether the initial identification of the AOI by BAGEL3 was based on the presence of bacteriocin-associated genes (context) or a specific bacteriocin structural gene

Potential Producer	Phylum	Class	BAGEL3 prediction
*Bifidobacterium longum* subsp. *infantis* JCM 1222	Actinobacteria	Lantibiotic	Context
*Bifidobacterium* sp. 12 1 47BFAA	Actinobacteria	Lantibiotic	BLD_1648
*Eggerthella* sp. HGA1	Actinobacteria	>10 kDa	Linocin M18
*Bacteroides dorei* DSM 17855	Bacteroidetes	Sactipeptide	Context
*Bacteroides fragilis* 3 1 12	Bacteroidetes	Sactipeptide	Context
*Bacteroides* sp. 2 1 16	Bacteroidetes	Lantibiotic	Manual
*Bacteroides* sp. 2 1 56FAA	Bacteroidetes	Unmodified	Manual
*Bacteroides* sp. 9 1 42FAA	Bacteroidetes	Sactipeptide	Context
*Bacteroides uniformis* ATCC 8492	Bacteroidetes	Sactipeptide	Context
*Anaerofustis stercorihominis* DSM 17244	Firmicutes	>10 kDa	Linocin M18
*Bacillus* sp. 7 6 55CFAA CT2	Firmicutes	>10 kDa	Colicin E9
*Bacillus* sp. 7 6 55CFAA CT2	Firmicutes	Lantibiotic	Haloduracin
*Dorea formicigenerans* 4 6 53AFAA	Firmicutes	Sactipeptide	Context
*Enterococcus faecalis* PC1.1	Firmicutes	>10 kDa	Enterolysin A
*Enterococcus faecalis* TX1302	Firmicutes	>10 kDa	Enterolysin A
*Enterococcus faecalis* TX1302	Firmicutes	Lantibiotic	Context
*Enterococcus faecalis* TX1341	Firmicutes	>10 kDa	Enterolysin A
*Enterococcus faecalis* TX1342	Firmicutes	>10 kDa	Enterolysin A
*Enterococcus faecalis* TX1342	Firmicutes	Lantibiotic	Context
*Enterococcus faecalis* TX1467	Firmicutes	>10 kDa	Enterolysin A
*Enterococcus faecalis* TX1467	Firmicutes	Lantibiotic	Context
*Enterococcus faecalis* TX2137	Firmicutes	>10 kDa	Enterolysin A
*Enterococcus faecalis* TX2137	Firmicutes	Lantibiotic	Context
*Enterococcus faecalis* TX4244	Firmicutes	>10 kDa	Enterolysin A
*Enterococcus faecalis* TX4244	Firmicutes	>10 kDa	Enterolysin A
*Holdemania filiformis* DSM 12042	Firmicutes	>10 kDa	Linocin M18
*Lactobacillus acidophilus* ATCC 4796	Firmicutes	>10 kDa	Enterolysin A
*Lactobacillus acidophilus* ATCC 4796	Firmicutes	>10 kDa	Helveticin J
*Lactobacillus antri* DSM 16041	Firmicutes	>10 kDa	Enterolysin A
*Lactobacillus brevis* subsp. *gravesensis* ATCC 27305	Firmicutes	Unmodified	Plantaricin NC8
*Lactobacillus delbrueckii* subsp. *lactis* DSM 20072	Firmicutes	>10 kDa	Enterolysin A
*Lactobacillus helveticus* DSM 20075	Firmicutes	>10 kDa	Helveticin J
*Lactobacillus helveticus* DSM 20075	Firmicutes	>10 kDa	Helveticin J
*Lactobacillus ultunensis* DSM 16047	Firmicutes	>10 kDa	Helveticin J
*Lactobacillus ultunensis* DSM 16047	Firmicutes	>10 kDa	Enterolysin A
*Lactobacillus ultunensis* DSM 16047	Firmicutes	>10 kDa	Helveticin J
*Listeria innocua* ATCC 33091	Firmicutes	LAP	Context
*Marvinbryantia formatexigens* DSM 14469	Firmicutes	Sactipeptide	Context
*Roseburia intestinalis* L1 82	Firmicutes	Sactipeptide	Context
*Ruminococcus obeum* A2 162	Firmicutes	Lantibiotic	Context
*Ruminococcus* sp. 5 1 39B FAA	Firmicutes	Lantibiotic	Context
*Streptococcus anginosus* 1 2 62CV	Firmicutes	Unmodified	Multiple
*Streptococcus infantarius* subsp. *infantarius* ATCC BAA 102	Firmicutes	Unmodified	Multiple
*Streptococcus* sp. 2 1 36FAA	Firmicutes	Class IIc	Context
*Fusobacterium ulcerans* ATCC 49185	Fusobacteria	>10 kDa	Linocin M18
*Fusobacterium varium* ATCC 27725	Fusobacteria	>10 kDa	Linocin M18
*Arcobacter butzleri* JV22	Proteobacteria	>10 kDa	Colicin E9
*Escherichia coli* MS 110 3	Proteobacteria	>10 kDa	Colicin
*Escherichia coli* MS 110 3	Proteobacteria	>10 kDa	Colicin
*Escherichia coli* MS 119 7	Proteobacteria	>10 kDa	Colicin
*Escherichia coli* MS 124 1	Proteobacteria	>10 kDa	Colicin
*Escherichia coli* MS 146 1	Proteobacteria	>10 kDa	Linocin M18
*Escherichia coli* MS 153 1	Proteobacteria	>10 kDa	Colicin
*Escherichia coli* MS 16 3	Proteobacteria	>10 kDa	Colicin
*Escherichia coli* MS 16 3	Proteobacteria	>10 kDa	Colicin
*Escherichia coli* MS 16 3	Proteobacteria	>10 kDa	Colicin
*Escherichia coli* MS 185 1	Proteobacteria	>10 kDa	Colicin E9
*Escherichia coli* MS 196 1	Proteobacteria	>10 kDa	Colicin-10
*Escherichia coli* MS 200 1	Proteobacteria	>10 kDa	Colicin
*Escherichia coli* MS 21 1	Proteobacteria	>10 kDa	Colicin
*Escherichia coli* MS 45 1	Proteobacteria	Microcin	Microcin H47
*Escherichia coli* MS 45 1	Proteobacteria	>10 kDa	Colicin
*Escherichia coli* MS 57 2	Proteobacteria	>10 kDa	Colicin E9
*Escherichia coli* MS 78 1	Proteobacteria	>10 kDa	Colicin
*Escherichia coli* MS 85 1	Proteobacteria	>10 kDa	Colicin E9
*Escherichia coli* SE11	Proteobacteria	>10 kDa	Colicin
*Escherichia* sp. 3 2 53FAA	Proteobacteria	>10 kDa	Colicin
*Klebsiella* sp. MS 92 3	Proteobacteria	>10 kDa	Klebicin B
*Providencia rettgeri* DSM 1131	Proteobacteria	>10 kDa	Colicin A
*Yokenella regensburgei* ATCC 43003	Proteobacteria	Microcin	Context
*Anaerobaculum hydrogeniformans* ATCC BAA 1850	Synergistetes	>10 kDa	Linocin M18
*Synergistes* sp. 3 1 syn1	Synergistetes	>10 kDa	Linocin M18

### Further analysis of PBGCs of particular interest

**Fig. 2:**
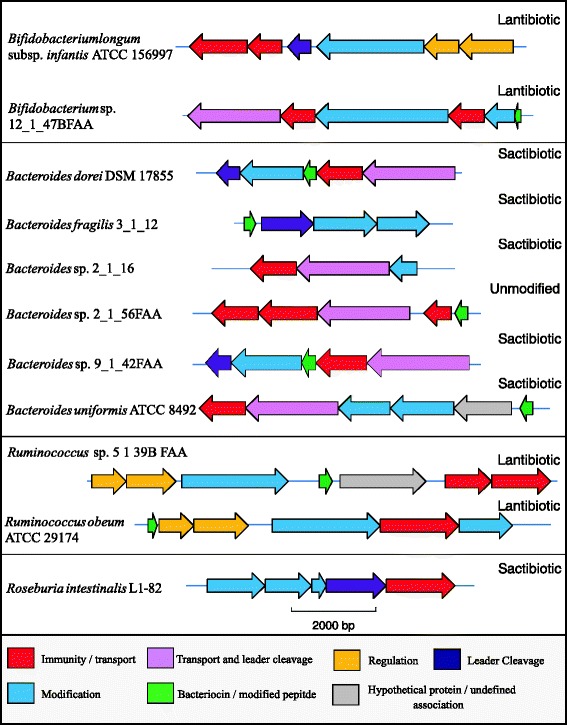
Diagrammatic representation of PBGCs deemed of particular interest

Sixty-three PBGCs are described in the Additional file 2: Supplementary Text and depicted in Additional file 3: Figures S1, Additional file 4: Figure S2 and Additional file 5: Figure S3. 11 PBGCs from 3 different phyla were deemed of particular interest and were selected for further *in silico* analysis based on the relative rarity with which bacteriocin production has been associated with the corresponding genus (*Bacteroides* and *Roseburia*), on the probiotic potential of strains from the genus (*Bifidobacterium*) or due to the importance/perceived importance of the genus in a gut environment (*Bacteroides, Roseburia, Ruminococcus*) (Fig. 2).

#### Identification of novel PBGCs in bifidobacteria

Bifidobacteria are an important group of human gut commensal bacteria, accounting for between 3 and 7 % of the gut microbiota in adults and up to 91 % in newborns [29]. Members of this genus have a long history of use as health-promoting/probiotic strains due to traits such as the regulation of intestinal microbial homeostasis, the inhibition of pathogens, the modulation of local and systemic immune responses, the maintenance of gastrointestinal barrier function, the production of vitamins and the bioconversion of a number of dietary compounds into bioactive molecules [30]. Bifidobacteria have the potential to suppress the growth of both Gram-negative and Gram-positive bacteria but, to date, this activity has been more often attributed to the inhibitory action of organic acids rather than bacteriocin production [31, 32]. For a review of the relatively rare examples of bacteriocin production by bifidobacteria see Martinez *et al*. [31]. Our *in silico* screen identified PBGCs of note in *Bifidobacterium longum* subsp. *infantis* ATCC 15697 and *Bifidobacterium* sp. 12_1_47BFA (Fig. 1).

*Bifidobacterium longum* subsp. *infantis* ATCC 15697 was isolated from human infant faeces and sequenced by the Joint Genome Institute (JGI) [33, 34]. A previous study has shown that this strain has the ability to reduce the levels of plasma endotoxins via modulation of the gut microbiota. However the authors concluded that the effect was mediated by increased levels of faecal organic acids [35]. The cluster of six genes identified are predicted to encode a LanL-type lantipeptide based on the presence of a LanL-type lanthionine synthetase gene. More specifically, the 8,139 bp cluster contains several lantibiotic-related genes including a putative lanthionine synthetase (conserved domain pfam05147 3.10e-10), a putative oligopeptidase (conserved domain pfam00326 5.24e-08) and a putative ABC transporter containing ATP-binding and permease subunits (conserved domains cd03255 and pfam02867 respectively). The cluster also contained a two-component regulatory system consisting of a putative histidine kinase (conserved domain CGO4585 6.70e-18) and a putative transcriptional response regulator (conserved domain COG2197 8.85e-57).

*Bifidobacterium* sp. 12_1_47BFA was recovered from inflamed biopsy tissue from a 25-year-old female patient with Crohn’s disease and its genome was found to contain a 7,996 bp lantibiotic cluster comprising six genes (Fig. 1). A putative lantibiotic prepeptide LanA was found to be similar to BLD_1648 (BAGEL3 bacteriocin I database 4e-43), a feature that was further supported by manual annotation (conserved domain TIGR03893 6.47e-9). Also present in the area of interest was a putative LanM lantibiotic biosynthesis protein (conserved domain cd04792 0.0), a putative multidrug ABC transporter ATP-binding protein putatively involved in lantibiotic immunity (conserved domain cd03230 8.53e-42) and an ABC-type bacteriocin/lantibiotic exporter (conserved domain COG2274 7.59e-145) significantly similar (BlastP 4e-117) to the *crnT* protein responsible for transport and leader cleavage of the bacteriocin carnolysin [36]. The area of interest also contained a FMN-dependent reductase (conserved domain pfam03358 5.13e-09) similar to that located within the carnolysin-associated *crnJ* protein [36]. This family of proteins has been suggested to be an atypical lantibiotic post-translational modification protein [20, 37].

#### *Identification of novel PBGCs in* Bacteroides *spp.*

*Bacteroides* are Gram-negative, non-spore-forming, obligate anaerobes and near universal constituents of the human gut microbiota, especially prevalent in those individuals whose long-term diets are rich in protein and animal fat [38]. Translocation from the GIT can however result, in some cases, in bacteraemia and abscess formation [39]. Weight loss in obese humans subjected to dietary or surgical intervention has been associated with increased relative abundance in the phylum Bacteroidetes, with specific members including *Bacteroides* spp., *Bacteroides-Prevotella* spp. or the *Bacteroides fragilis* group bacteria having been associated with this phenomenon [40–43]. Despite their importance as a human gut commensal, there have been relatively few reports of bacteriocin production by members of the *Bacteroides* to date [44–47]. In this study, six PBGCs were identified in *Bacteroides* strains that possessed features typical of sactipeptide (4), lantibiotic (1) or unmodified bacteriocin (1) clusters.

*Bacteroides dorei* has been observed to be common in patients with active coeliac disease and it has also been proposed that the species be used as an indicator of water contamination by human faecal material [48, 49]. *B. dorei* DSM 17855 was isolated from a healthy, 23 year old, Japanese male [50] and its genome was found to contain a five gene, 5,711 bp sactipeptide-like gene cluster (Fig. 1). The cluster contained genes encoding a putative ABC-type transporter ATP-binding protein (BlastP 0.0, conserved domain COG2274 3.02e-34), a putative hemolysin secretion protein HlyD (BlastP 0.0), a structural gene belonging to pfam family pf10439 (Bacteriocin class II with double-glycine leader peptide), a radical SAM domain-containing protein hypothesised to be involved in peptide modification (conserved domain TIGR03962 1.46e-06) and a putative bacteriocin-associated C39 family peptidase (conserved domain pfam03412 1.13e-11). The latter may be involved in transport across the membrane in addition to leader cleavage, either alone or in conjunction with HlyD.

*Bacteroides fragilis*-produced metabolites are important in the activation and regulation of the T-cell-dependent immune response [39, 51] and its administration as a therapeutic has been proposed for gastrointestinal and behavioural symptoms associated with human neurodevelopmental disorders [52]. The genome of *B. fragilis* 3_1_12 found to contain a four gene, 4267 bp sactipeptide-like cluster (Fig. 1). The putative structural gene belongs to pfam family PF14406 (Ribosomally synthesized peptide in Bacteroidetes) and BlastP identified it as a putative bacteriocin-type signal sequence containing a predicted leader sequence associated with peptide modification (conserved domain TIGR04149 1.34e-12). Immediately downstream is a putative lipoprotein belong to pfam family PF08139 followed by a pair of putative radical SAM proteins, predicted to be involved in peptide modification. These radical SAM proteins, members of families TIGR04085 and TIGR04150, respectively, are known to occur in cassettes together with the bacteriocin signal sequence noted above [53].

*Bacteroides* sp. 2_1_16 was isolated from a healthy biopsy of the descending colon of a 58-year old female patient undergoing colonoscopy its genome was found to contain a 4,167 bp, three-gene cluster predicted to be sactipeptide-encoding based on the presence of a SacCD homolog (Fig. 1). However, manual annotation also revealed a cluster of several genes with homology with those typically associated with lantibiotic production. Specifically, the cluster contained a putative LanC-like lanthionine synthetase (conserved domain cd04793 6.02e-08), a putative ABC transporter predicted to be a bacteriocin/lantibiotic transporter based on conserved domains (COG2274 0.0) and a putative ABC transporter secretion protein closely related to hemolysin secretors (conserved domain TIGR01843 1.86e-22). However, a putative structural peptide-encoding gene could not be identified in this gene cluster.

The genome of *Bacteroides* sp. 2_1_56FAA was found to possess a 6,069 bp cluster containing five genes of note (Fig. 1). Manual annotation revealed a gene predicted to encode a ribosomally synthesised peptide (pfam PF14406 0.00024 [54]), located immediately upstream of a putative CAAX protease self-immunity family determinant (conserved domain pfam02517 8.17e-11). A gene encoding a putative ABC transporter containing a C39B peptidase domain (COG2274 7.75e-159), predicted to be responsible for transport and leader cleavage, was also present. Two additional possible transport genes were identified immediately downstream, both putative hemolysin secretion proteins (conserved domain pfam13437 5.74e-09 and conserved domain pfam13437 5.37e-11, respectively). The lack of any bacteriocin-modification genes suggests that this cluster encodes an unmodified bacteriocin.

*Bacteroides* sp. 9_1_42FAA was isolated from the duodenum of a 47 year old female patient and its genome contained a 5,714 bp area of interest comprised five genes, This cluster was identified as a potential sactipeptide based on the presence of a SacCD homolog (Fig. 1). The structural peptide putatively encoded within this cluster also possesses features associated with pfam family PF10439.4 i.e. unmodified subclass IIc bacteriocins. The area of interest also contains a putative ABC-type bacteriocin/lantibiotic exporter (contains conserved domain COG2274 0.0), a putative hemolysin secretion family protein (conserved domain TIGR01843 3.45e-06), a putative radical SAM peptide modification protein (conserved domain TIGR03962 1.47e-17), and a putative bacteriocin transporter containing an endopeptidase C39 domain (potentially involved in bacteriocin preprocessing; conserved domain pfam03412 1.13e-11) [55]. This sequence exhibited very high (99 %) nucleotide identity to the aforementioned gene cluster in *B. dorei* DSM 17855. This similarity includes structural genes with 100 % amino acid sequence identity.

It has been previously documented that orally administering *Bacteroides uniformis* (strain CECT 7771) ameliorated high fat diet-induced metabolic and immune dysfunction associated with an altered gut microbiota in adult C57BL-6 mice [56]. Inspection of the genome of *B. uniformis* ATCC 8492 revealed a 7,976 bp, five-gene sactipeptide-like cluster (Fig. 1). Manual annotation identified a putative bacteriocin-type signal sequence containing a conserved TIGR04149 domain (7.43e-09). The area of interest also contained a pair of putative peptide-modifying radical SAM proteins (conserved domains TIGR04148 and TIGR04150 respectively) similar to those in *B. fragilis* 3_1_12 that were referred to above, a putative ABC-type bacteriocin exporter (conserved domain COG2274 0.0) and a putative hemolysin secretion protein (conserved domain pfam13437 1.02e-16).

#### *Identification of novel PBGCs in* Ruminococcus *spp.*

Ruminococci are Gram-positive anaerobes commonly found in the human gut, where they have been proposed to play a pivotal role in the fermentation of resistant starch [57]. There have been several previous reports of bacteriocin production by members of the ruminococci, including a class IIa lantibiotic, ruminococcin A, produced by *Ruminococcus gnavus* E1 and two distinct class III bacteriocins produced by *Ruminococcus albus* 7 [58–60]. We identified two apparently novel *Ruminococcus*-associated PBGCs, from among a total of 35 Firmicutes-associated clusters (Additional file 2: Supplementary Text).

The genome of *Ruminococcus* sp. 5_1_39_B_FAA contained a 13,553 bp lantibiotic-like cluster containing six genes (Fig. 1). The cluster contained a putative response regulator receiver protein (conserved domain COG3279 3.95e-24), a putative histidine kinase (conserved domain pfam14501 3.5e-20), a putative type 2 lantibiotic biosynthesis protein LanM (conserved domain TIGR03897 0.0), a putative UviB-like bacteriocin (BAGEL3 bacteriocin II database 3e-11), a putative ABC transporter ATP-binding protein (conserved domain COG1136 8.20e-111) and a putative efflux ABC transporter permease protein.

Strains of *Ruminococcus obeum* have been shown to restrict *Vibrio cholerae* infection *via* a quorum-sensing-mediated mechanism [61]. *Ruminococcus obeum* ATCC 29174 was isolated from human faeces and sequenced by the Washington University Genome Sequencing Centre. A 8,879 bp lantibiotic-like cluster comprising six genes was identified (Fig. 1). The putative structural gene was found to resemble geobacillin I (BAGEL3 bacteriocin I database 5e-12), a nisin homolog isolated from *Geobacillus thermodenitrificans* [62]. Also present in the area of interest were genes that appear to encode a two-component regulatory system, consisting of a putative histidine kinase (conserved domain COG0642 1.84e-24) and a putative *NisR* homolog containing signal receiver and effector domains (cd00156 and cd00383 respectively). Furthermore, genes potentially enoding a lantibiotic dehydratase similar to the entianin (lantibiotic) modification protein *EtnB* (BlastP 0.0) [63], an ABC transport protein similar to SpaT (transportation of the lantibiotic subtilin; BlastP 0.0) and a lanthionine synthetase protein similar to SpaC (modification of subtilin; BlastP 6e-117) were identified.

#### *Identification of a novel PBGC in* Roseburia *spp.*

*Roseburia* is a genus of Gram positive, butyrate-producers found to be negatively associated with type 2 diabetes and ulcerative colitis [64, 65]. It has also been linked with ameliorating high-fat diet induced metabolic alterations in mice [66]. The only *Roseburia*-associated bacteriocin-producer to have been identified to date is *Roseburia faecis* M72/1 [67]. *Roseburia intestinalis* L1-82, the type strain, was found to contain a five gene, 6078 bp sactipeptide-like cluster (Fig. 1). The area of interest contained a putative bacteriocin-associated radical SAM protein (conversed domain TIGR04068 0.0), a putative peptide maturation system protein (conserved domain TIGR04066 8.58e-165), a putative peptide maturation system acyl carrier-related protein (conserved domain TIGR04069 1.15e-29), a subtilase family serine protease (conserved domain cd07492 7.11e-40) and a putative ABC transporter (conserved domain cd03228 5.95e-65). However, there were no immediately obvious bacteriocin structural or immunity genes in the area of interest and so it is particularly unclear if this cluster has the potential to produce an antimicrobial.

## Conclusions

The large number of fully sequenced genomes available in public repositories means that genome-mining approaches are increasingly valuable with respect to the identification of novel genes and gene clusters [68–70]. As it has already been established that *in silico* approaches can be applied to the human microbiome for the purpose of identifying antimicrobial-producing microorganisms [25, 71], and that bacteriocins identified in this manner can be produced *in vitro* [18], it is apparent that there are considerable potential benefits in screening for and harnessing putative bacteriocin gene clusters from such databases.

It is commonly reported that between 30 and 99 % of bacteria have the potential to produce at least one bacteriocin [72, 73]. It is thus notable that this *in silico*-based study identified just 59 genomes encoding probable PBGCs from 382 reference genomes, a frequency of just 15.4 %. It is unclear whether this low number is representative of bacteriocin-production in the human GIT or an underestimation due to biases in identification of gene clusters. In support of the former of these theories, a recent study on the human microbiome by Zheng *et al*. reported that the gut contained the lowest density of putative bacteriocin genes of all body sites investigated [25]. That study identified 123 putative lantibiotic, 56 putative class II bacteriocin and 148 putative class III bacteriocin gene clusters in the gut environment. Interestingly, only one sactipeptide of gut origin, a subtilosin A, was reported by Zheng *et al*. [25]. The discrepancy between the results reported by this study and those reported by Zheng *et al.* can be explained by differences in methodology. This method used BAGEL3 for the initial analysis while Zheng *et al.* performed a PSI-BLAST-based approach using the amino acid sequences from the BAGEL3 bacteriocin database as driver sequences. Furthermore, we manually annotated the potential clusters returned initially, resulting in a dramatic decrease in reported PBGCs. It is noteworthy that *in silico* screens are limited by their dependence on similarity to previously described bacteriocin-associated genes, meaning that is it possible to overlook completely novel bacteriocin clusters.

The vast majority of known/characterised lantibiotics are produced by members of the Firmicutes [74]. Similarly, of the 11 lantibiotic PBGCs identified in this study, seven were found in the genomes of Firmicutes, with two associated with bifidobacteria (Actinobacteria) and two with *Bacteroides* spp. (Bacteroidetes). While these clusters typically contained features that are common to lantibiotic-associated gene clusters, two putative lantibiotic clusters (in *Bifidobacterium* sp. 12_1_47BFAA and *Enterococcus faecalis* TX1342 (Additional file 2: Supplementary Results; Additional file 3: Figures S1 and Additional file 5: Figure S3 respectively)) contained predicted FMN reductase genes in addition to those more traditionally associated with lantibiotic modification.

It is apparent that the *in silico* screen identified gene clusters representative of some classes of bacteriocin more frequently than others. Clusters resembling those associated with the production of bacteriolysins (formerly referred to as class III bacteriocins) were most common. The large numbers of colicin-like and enterolysin A-like clusters was possibly due to the overrepresentation of *E. coli* in the reference genome database and the relative ease of detection. It appears that enterolysin A does not possess a specific immunity gene; instead, resistance results from the absence of specific binding receptors [75], making this single gene potentially easier to detect than a multi-gene operon. On the other hand, the relatively low frequency of class II bacteriocins (three unmodified and one class IIc) cannot be explained in a similar manner. It is unclear whether this paucity is due to the methodology or an actual scarcity of class II bacteriocin producers in the gut microbiota. Comparatively, Zheng *et al*. identified 56 class II bacteriocin structural genes from gut-associated strains [25] suggesting that either this is an overestimation due to the lack of manual annotation or the approach used in this study is not ideal for the identification of Class II bacteriocins.

In several cases, complete gene clusters were identified that lacked an obvious bacteriocin structural gene. Compared to other classes, the number of described and characterised sactipeptides is relatively small so it may be possible that BAGEL3 and the nr database do not contain any homologs of the structural proteins encoded by *Bacteroides* sp. 2_1_16 and *Roseburia intestinalis* L1-82. This may also explain the relatively low incidence of sactipeptides reported by Zheng *et al.* [25]. The putative lantibiotic cluster identified in *Bifidobacterium longum* subsp. *infantis* ATCC 156997 was also missing an obvious structural gene but may be explained by the same hypothesis, as it is a potential LanL-type lantibiotic, a subclass which contains only one previously described member Venezuelin [76].

This comprehensive *in silico* study led to the identification of PBGCs in species not previously associated with bacteriocin production, for example *Bacteroides uniformis* and *Roseburia intestinalis.* We also identified potential bacteriocin gene clusters in two *Bifidobacterium* species, a genus which has long been thought of as beneficial to the human host. It is not possible, by *in silico* methods alone, to state conclusively if these bacteriocins are produced *in vitro*. However, if even a portion of these gene clusters are responsible for bacteriocin production in the corresponding strain, it could greatly expand the arsenal of bacteriocins available for use in food and healthcare. Such investigations will be the focus of our future studies.

## Methods

### Initial screening of reference genomes for bacteriocin gene clusters

The GIT subset (382 available sequences as of 20/11/2014) of the HMP’s reference genome database (http://www.hmpdacc.org/HMRGD/) was downloaded in multi-FASTA format and both complete and draft genomes were screened for putative bacteriocin gene clusters using the web-version of BAGEL3 (http://bagel2.molgenrug.nl/index.php/bagel3).

### Further investigation of individual gene clusters

Approximately 20 kb of sequence data containing the gene/genes identified as being of potential interest by BAGEL3 were extracted and the sequences were manually annotated using the software ARTEMIS [77]. Predicted coding regions were analysed using the BlastP web server on NCBI (http://www.ncbi.nlm.nih.gov/BLAST) and the nr database. The coding regions were also analysed for the presence of conserved domains and, where applicable, compared to previously described gene clusters using the Artemis Comparison Tool (ACT) [78].

## Additional files

Additional file 1: Table S1. List of the 130 unique putative producers identified by BAGEL3 (DOCX 15 kb) Additional file 2: Supplementary Text. Description of remaining 63 PBGCs not covered in main text (DOCX 39 kb) Additional file 3: Figure S1. Diagrammatic representation of remaining PBGCs identified in the Actinobacteria, Fusobacteria and Synergistetes (DOCX 25 kb) Additional file 4: Figure S2. Diagrammatic representation of remaining PBGCs identified in the Firmicutes (DOCX 62 kb) Additional file 5: Figure S3. Diagrammatic representation of remaining PBGCs identified in the Proteobacteria (DOCX 52 kb)

## Abbreviations

ACT: Artemis Comparison Tool
AOI: Area of Interest
GI: Gastrointestinal
GIT: Gastrointestinal Tract
HMP: Human Microbiome Project
JGI: Joint Genome Institute
LAP: Linear Azol(in)e-containing Peptide
PBGC: Putative Bacteriocin Gene Cluster
