# Supplementary material for: In silico identification of bacteriocin gene clusters in the gastrointestinal tract, based on the Human Microbiome Project’s reference genome database
Source: BMC Microbiol. 2015 Sep 16;15:183. doi: 10.1186/s12866-015-0515-4 (PMC4573289; doi:10.1186/s12866-015-0515-4)
Supplement: Additional file 1: Table S1. — List of the 130 unique putative producers identified by BAGEL3 (DOCX 15 kb) [file 12866_2015_515_MOESM1_ESM.docx]

Table S1. 130 unique putative producers identified by BAGEL3

| Producer |
| --- |
| Bifidobacterium_longum_subsp_infantis_JCM_1222 |
| Bifidobacterium_sp_12_1_47BFAA |
| Collinsella_stercoris_DSM_13279 |
| Eggerthella_sp_HGA1 |
| Propionibacterium_sp_5_U_42AFAA |
| Bacteroides_dorei_DSM_17855 |
| Bacteroides_fragilis_3_1_12 |
| Bacteroides_sp_2_1_16 |
| Bacteroides_sp_2_1_56FAA |
| Bacteroides_sp_9_1_42FAA |
| Bacteroides_uniformis_ATCC_8492 |
| Odoribacter_laneus_YIT_12061 |
| Anaerofustis_stercorihominis_DSM_17244 |
| Anaerotruncus_colihominis_DSM_17241 |
| Bacillus_sp_7_6_55CFAA_CT2 |
| Blautia_hansenii_DSM_20583 |
| Butyrivibrio_crossotus_DSM_2876 |
| Catenibacterium_mitsuokai_DSM_15897 |
| Clostridiales_sp_SSC_2 |
| Coprobacillus_sp_29_1 |
| Coprobacillus_sp_8_2_54BFAA |
| Coprobacillus_sp_D6 |
| Coprococcus_catus_GD_7 |
| Desulfitobacterium_hafniense_DP7 |
| Dorea_formicigenerans_4_6_53AFAA |
| Dorea_longicatena_DSM_13814 |
| Enterococcus_faecalis_PC1.1 |
| Enterococcus_faecalis_TX0104 |
| Enterococcus_faecalis_TX1302 |
| Enterococcus_faecalis_TX1341 |
| Enterococcus_faecalis_TX1342 |
| Enterococcus_faecalis_TX1346 |
| Enterococcus_faecalis_TX1467 |
| Enterococcus_faecalis_TX2134 |
| Enterococcus_faecalis_TX2137 |
| Enterococcus_faecalis_TX4244 |
| Enterococcus_faecium_PC41 |
| Enterococcus_faecium_TX1330 |
| Erysipelotrichaceae_bacterium_3_1_53 |
| Erysipelotrichaceae_bacterium_5_2_54FAA |
| Erysipelotrichaceae_bacterium_6_1_45 |
| Eubacterium_hallii_DSM_3353 |
| Eubacterium_siraeum_DSM_15702 |
| Flavonifractor_plautii_ATCC_29863 |
| Holdemania_filiformis_DSM_12042 |
| Lachnospiraceae_bacterium_2_1_46FAA |
| Lachnospiraceae_bacterium_2_1_58FAA |
| Lachnospiraceae_bacterium_3_1_57FAA_CT1 |
| Lachnospiraceae_bacterium_5_1_63FAA |
| Lachnospiraceae_bacterium_7_1_58FAA |
| Lactobacillus_acidophilus_ATCC_4796 |
| Lactobacillus_antri_DSM_16041 |
| Lactobacillus_brevis_subsp_gravesensis_ATCC_27305 |
| Lactobacillus_delbrueckii_subsp_lactis_DSM_20072 |
| Lactobacillus_helveticus_DSM_20075 |
| Lactobacillus_plantarum_subsp_plantarum_ATCC_14917 |
| Lactobacillus_reuteri_CF48_3A |
| Lactobacillus_reuteri_JCM_1112 |
| Lactobacillus_reuteri_MM2_3 |
| Lactobacillus_reuteri_MM4_1A |
| Lactobacillus_reuteri_SD2112 |
| Lactobacillus_rhamnosus_ATCC_21052 |
| Lactobacillus_ultunensis_DSM_16047 |
| Listeria_innocua_ATCC_33091 |
| Marvinbryantia_formatexigens_DSM_14469 |
| Paenibacillus_sp_HGF7_contig00140 |
| Paenibacillus_sp_HGF7_contig00161 |
| Paenibacillus_sp_HGF7_contig00230 |
| Parvimonas_micra_ATCC_33270 |
| Pediococcus_acidilactici_7_4 |
| Roseburia_intestinalis_L1_82 |
| Ruminococcus_obeum_A2_162 |
| Ruminococcus_obeum_ATCC_29174 |
| Ruminococcus_sp_5_1_39B_FAA |
| Ruminococcus_sp_SR1_5 |
| Streptococcus_anginosus_1_2_62CV |
| Streptococcus_infantarius_subsp_infantarius_ATCC_BAA_102 |
| Streptococcus_sp_2_1_36FAA |
| Fusobacterium_sp_D12 |
| Fusobacterium_ulcerans_ATCC_49185 |
| Fusobacterium_varium_ATCC_27725 |
| Arcobacter_butzleri_JV22 |
| Campylobacter_upsaliensis_JV21 |
| Citrobacter_freundii_4_7_47CFAA |
| Citrobacter_sp_30_2 |
| Citrobacter_youngae_ATCC_29220 |
| Desulfovibrio_sp_3_1_syn3 |
| Desulfovibrio_sp_6_1_46AFAA |
| Edwardsiella_tarda_ATCC_23685 |
| Enterobacter_cancerogenus_ATCC_35316 |
| Enterobacter_cloacae_subsp_cloacae_NCTC_9394 |
| Enterobacteriaceae_bacterium_9_2_54FAA |
| Escherichia_coli_4_1_47FAA |
| Escherichia_coli_MS_107_1 |
| Escherichia_coli_MS_110_3 |
| Escherichia_coli_MS_115_1 |
| Escherichia_coli_MS_116_1 |
| Escherichia_coli_MS_117_3 |
| Escherichia_coli_MS_119_7 |
| Escherichia_coli_MS_124_1 |
| Escherichia_coli_MS_146_1 |
| Escherichia_coli_MS_153_1 |
| Escherichia_coli_MS_16_3 |
| Escherichia_coli_MS_175_1 |
| Escherichia_coli_MS_185_1 |
| Escherichia_coli_MS_187_1 |
| Escherichia_coli_MS_196_1 |
| Escherichia_coli_MS_198_1 |
| Escherichia_coli_MS_200_1 |
| Escherichia_coli_MS_21_1 |
| Escherichia_coli_MS_45_1 |
| Escherichia_coli_MS_57_2 |
| Escherichia_coli_MS_69_1 |
| Escherichia_coli_MS_78_1 |
| Escherichia_coli_MS_79_10 |
| Escherichia_coli_MS_85_1 |
| Escherichia_coli_SE11 |
| Escherichia_sp_1_1_43 |
| Escherichia_sp_3_2_53FAA |
| Escherichia_sp_4_1_40B |
| Helicobacter_bilis_ATCC_43879 |
| Klebsiella_sp_MS_92_3 |
| Oxalobacter_formigenes_OXCC13 |
| Proteus_penneri_ATCC_35198 |
| Providencia_alcalifaciens_DSM_30120 |
| Providencia_rettgeri_DSM_1131 |
| Providencia_rustigianii_DSM_4541 |
| Yokenella_regensburgei_ATCC_43003 |
| Anaerobaculum_hydrogeniformans_ATCC_BAA_1850 |
| Synergistes_sp_3_1_syn1 |
