# Supplementary material for: In silico identification of bacteriocin gene clusters in the gastrointestinal tract, based on the Human Microbiome Project’s reference genome database
Source: BMC Microbiol. 2015 Sep 16;15:183. doi: 10.1186/s12866-015-0515-4 (PMC4573289; doi:10.1186/s12866-015-0515-4)
Supplement: Additional file 2: Supplementary Text. — Description of remaining 63 PBGCs not covered in main text (DOCX 39 kb) [file 12866_2015_515_MOESM2_ESM.docx]

**Supplementary Results**

**Actinobacteria-associated PBGCs**

***Eggerthella* sp. HGA1** – This genome contained a putative linocin M18-like structural protein (BAGEL3 bacteroicin III database 5e-22, BlastP 3e-95) (Figure S1). Linocin M18 is a high molecular weight bacteriocin first isolated from *Brevibacterium linens* M18 with an antilisterial effect [[1](#_ENREF_1)].

**Firmicutes-associated PBGCs**

***Anaerofustis stercorihominis* DSM 17244** – This genome contained a putative linocin M18-like structural protein (BAGEL3 bacteriocin III database Linocin M18 9e-25, BlastP 5e-107) (Figure S2).

***Bacillus* sp. 7 6 55CFAA** – This genome contains a 6 gene, 9086 bp class II lantibiotic cluster (Figure S2). There were two putative structural genes identified (BAGEL3 bacteriocin I database Haloduracin alpha 4e-13, BlastP lantibiotic lichenicidin A1 2e-06; BAGEL3 bacteriocin I database Haloduracin beta 6e-10, BlastP lantibiotic cytolysin 1e-06) [[2](#_ENREF_2)]. Immediately upstream are bacteriocin-related genes, including a putative LanM-like lantibiotic modification protein (BlastP 0.0), a putative LanT-like lantibiotic transport protein (BlastP 0.0, conserved domain COG2274 0.0), a putative AraC-family transcriptional regulator (BlastP 3e-103), and a putative S8 family peptidase (BlastP 0.0) containing a lantibiotic-specific protease domain (conserved domain cd07482 9.58e-102). However, no immunity gene was apparent.

A 2 gene, colicin-like gene cluster was also identified in this strain (Figure S2). This was comprised of a putative colicin E9 structural gene (BAGEL3 bacteriocin III database 1e-24, conserved domain pfam12639 1.01e-28) and a putative colicin immunity protein (BlastP 9e-26, conserved domain pfam01320 1.46e-06).

***Dorea formicigenerans* 4 6 53AFAA** – This genome contains an 8 gene, 8423 bp sactipeptide-like cluster (Figure S2). Manual annotation revealed genes putatively encoding a two-component regulatory system consisting of a putative VanS-like histidine kinase (BlastP 1e-180, conserved domain COG0642 2.13e-44) and a putative VanR-like transcriptional response regulator (BlastP 3e-167, conserved domain TIGR02154 2.61e-63), a putative ABC transporter ATP-binding protein (BlastP 0.0, conserved domain COG1131 8.06e-86), a putative ABC-2 transporter family protein (BlastP 7e-141, conserved domain pfam12730 4.23e-08) and a putative radical SAM protein (BlastP 0.0) predicted to be involved in bacteriocin maturation (conserved domain TIGR04068 8.67e-81). The gene predicted to encode the putative structural prepeptide identified returned no significant BlastP hits but manual annotation identified a gene encoding a putative bacteriocin precursor (conserved domain TIGR04067 2.11e-10). This area of interest also contained a putative lantibiotic ABC transporter ATP-binding protein (BlastP 1e-131) and a putative VanZ-like teicoplanin resistance gene (BlastP 2e-41, conserved domain pfam04892 3.16e-15).

***Enterococcus faecalis* PC1.1** – This genome contained a 2 gene enterolysin A cluster (Figure S2) comprising genes predicted to encode a puatative M23 family peptidase (BAGEL3 bacteriocin III database Enterolysin A 0.0, BlastP 0.0, conserved domain pfam01551 5.83e-13) and a putative bacteriocin-associated C39a family peptidase (conserved domain cd02549 1.48e-06).

***Enterococcus faecalis* TX1302** – This genome contained a 2 gene enterolysin A cluster (Figure S2) comprising genes predicted to encode a putative M23 peptidase (BAGEL3 bacteriocin III database Enterolysin A 0.0, BlastP 0.0, conserved domain pfam01551 5.83e-13) and a putative bacteriocin-associated C39a family peptidase (conserved domain cd02549 1.48e-06).

The genome also contains a 6 gene, 7848 bp cytolysin cluster (Figure S2), containing a pair of lantibiotic structural genes; CylLl (BAGEL3 bacteriocin II database Cytolysin_ClyLl 4e-45, BlastP 3e-09, conserved domain TIGR03893 4.78e-09) and ClyLs (BAGEL3 bacteriocin II database Cytolysin_ClyLs 1e-42, BlastP 3e-36, conserved domain TIGR03893 1.79e-14). The cluster also contained genes encoding a lantibiotic biosynthesis protein ClyM (BlastP 0.0, conserved domain cd04792 0.0), a cytolysin transport protein CylB (BlastP 0.0, conserved domain COG2274 2.18e-151), a ClyA serine protease responsible for lantibiotic leader peptide processing (BlastP 0.0, conserved domain cd07482 4.43e-54) and a cytolysin immunity protein CylI (BlastP 0.0).

***Enterococcus faecalis* TX1341** – This genome contained a 2 gene entrolysin A cluster (Figure S2) comprising genes predicted to encode a putative M23 peptidase (BAGEL3 bacteriocin III database Enterolysin A 0.0, BlastP 0.0, conserved domain pfam01551 6.56e-12) and a putative C39a family peptidase predicted to be involved in bacteriocin-processing (conserved domain cd02549 1.48e-06).

***Enterococcus faecalis* TX1342** – This genome contained a 2 gene entrolysin A cluster (Figure S2) comprising genes predicted to encode a putative M23 peptidase (BAGEL3 bacteriocin III database Enterolysin A 0.0, BlastP 0.0, conserved domain pfam01551 5.83e-13) and a putative C39a family peptidase predicted to be involved in bacteriocin-processing (conserved domain cd02549 1.17e-05).

The genome also contained a 6 gene, 6425 bp lantibiotic-like cluster (Figure S2) containing a pair of putative lantibiotic structural genes (BlastP 5e-05, conserved domain TIGR03893 4.99e-12; BlastP BAGEL3 bacteriocin I database BLD_1648 4e-06, conserved domain TIGR03893 5.71e-17). The cluster also contained genes predicted to encode a pair of putative LanM type 2 lantibiotic biosynthesis proteins (BlastP 0.0, conserved domain cd04792 9.78e-88; BlastP 0.0, conserved domain cd04792 1.14e-86), a putative FMN reductase (BlastP 3e-37, conserved domain pfam03358 3.21e-19) and a putative ABC-type lantibiotic transport protein (BlastP 0.0, conserved domain COG2274 3.49e-168).

***Enterococcus faecalis* TX1467** – The genome contains a 7 gene, 7845 bp cytolysin cluster (Figure S2) containing a pair of lantibiotic structural genes; a putative ClyLl (Bagel3 bacteriocin II database Cytolysin_ClyLl 1e-44, BlastP 2e-15, conserved domain TIGR03893 3.28e-09) and ClyLs (Bagel3 bacteriocin II database Cytolysin_ClyLs 1e-42, BlastP 3e-36, conserved domain TIGR03893 1.79e-14). The cluster also contained genes predicted to encode a pair of ClyM lantibiotic biosynthesis proteins (BlastP 0.0, conserved domain cd04792 1.10e-167; BlastP 0.0, cd04792 6.27e-44), a ClyB lantibiotic ABC transporter (BlastP 0.0, conserved domain COG2274 9.23e-150), a ClyA lantibiotic leader peptide processing serine protease (BlastP 0.0, conserved domain cd07482 3.58e-36) and a putative CylI cytolysin immunity protein (BlastP 0.0).

The genome also contains a 2 gene entrolysin A cluster (Figure S2) comprising genes predicted to encode a putative M23 peptidase (BAGEL3 bacteriocin III database Enterolysin A 0.0, BlastP 0.0, conserved domain pfam01551 5.83e-13) and a putative C39a family peptidase predicted to be involved in bacteriocin processing (conserved domain cd02549 1.48e-06).

***Enterococcus faecalis* TX2137** - This genome contained a 2 gene enterolysin A cluster (Figure S2) comprising genes predicted to encode a putative M23 peptidase (BAGEL3 bacteriocin III database Enterolysin A 0.0, BlastP 0.0, conserved domain pfam01551 5.83e-13) and a putative C39a family peptidase predicted to be involved in bacteriocin processing (conserved domain cd02549 2.10e-06).

The genome also contained a 5 gene, 6891 bp cytolysin cluster (Figure S2) containing a pair of putative lantibiotic structural genes; ClyLl (Bagel3 bacteriocin II database Cytolysin_ClyLl 4e-45, BlastP 2e-15, conserved domain TIGR03893 4.78e-09) and ClyLs (Bagel3 bacteriocin II database Cytolysin_ClyLs 1e-42, BlastP 2e-36, conserved domain TIGR03893 1.79e-14). The cluster also contained genes predicted to encode a ClyM lantibiotic biosynthesis protein (BlastP 0.0, conserved domain cd04792 0.0), a ClyB lantibiotic ABC transporter (BlastP 0.0, conserved domain COG2274 2.05e-151) and a ClyA lantibiotic leader peptide processing serine protease (BlastP 0.0, conserved domain cd07482 6.30e-55). Unlike the cytolysin clusters referred to above, there was no immunity protein present.

***Enterococcus faecalis* TX4244** - This genome contained two possible enterolysin A clusters. The first was a 2 gene cluster (Figure S2) comprising genes predicted to encode a putative Enteroloysin A structural protein (BAGEL3 bacteriocin III database 1e-178, conserved domain pfam01551 6.90e-12) and a putative C39a family peptidase predicted to be involved in bacteriocin processing (conserved domain cd02549 9.73e-06).

The second was also a 2 gene cluster (Figure S2) comprising genes predicted to encode a a putative Enteroloysin A structural protein (BAGEL3 bacteriocin III database 0.0, conserved domain pfam01551 5.83e-13) and a putative C39a family peptidase predicted to be involved in bacteriocin processing (conserved domain cd02549 1.48e-06).

***Holdemania filiformis* DSM 12042 ­**– This genome contained a putative linocin M18-like structural gene (BAGEL3 bacteriocin III database Linocin M18 5e-25, BlastP 4e-88) (Figure S2).

***Listeria innocua* ATCC 33091** – This genome contained an 8 gene, 5891 bp area of interest (Figure S2). This cluster contained genes predicted to encode a listeriolysin S-like prepeptide (BlastP 7e-23), an putative ABC transport system with both ATP-binding and permease subunits (BlastP 0.0 conserved domain pfam00005 5.21e-20; 6e-176 respectively), a hypothetical protein predicted by BAGEL3 to be a phosphotransferase (PF02378.13 5.7e-05), a putative SagB-type dehydrogenase domain-containing protein (BlastP 5e-156, conserved domain TIGR03605 8.53e-67), a putative SagC family bacteriocin biosynthesis cyclodehydratase (BlastP 0.0, conserved domain TIGR03603 2.03e-92), a putative SagD family bacteriocin-associated docking protein (BlastP 0.0, conserved domain TIGR03604 1.53e-124), and a putative CAAX protease involved in bacteriocin immunity (BlastP 2e-128, PF02517 2e-9). This cluster had an overall architecture similar to the *Listeria* pathogenicity island LIPI-3 described by Clayton *et al*.[[3](#_ENREF_3)].

***Marvinbryantia formatexigens* DSM 14469** – This genome contained a 4 gene, 4643 bp sactipeptide cluster (Figure S2). There were no significant BlastP hits for the putative structural gene product identified by BAGEL3 but the area of interest also contained genes predicted to encode a putative radical SAM protein (BlastP 5e-18) containing a domain involved in bacteriocin maturation (TIGR04068 2.24e-16), a putative ABC transporter permease (BlastP 2e-43) and a putative ABC transporter ATP-binding protein (BlastP 3e-58) The latter two genes were also predicted by BAGEL3 to be involved in bacteriocin/lantibiotic export.

***Streptococcus anginosus* 1 2 62CV** – This genome contained a 7244 bp area of interest containing 9 bacteriocin-related genes (Figure S2). This area of interest included genes predicted to encode a putative CAAX protease self-immunity family protein (BlastP 2e-137, conserved domain pfam02517 8.54e-12), a putative class II bacteriocin structural protein protein (BlastP 3e-40, conserved domain pfam10439 5.49e-03), a putative bacteriocin self-immunity protein (BlastP 5e-9), a predicted Bovicin 255 variant structural protein (BAGEL3 bacteriocin II database 1e-25), a putative bacteriocin self-immunity protein (BlastP 6e-10), another putative bovicin 255 variant structural protein (BAGEL3 bacteriocin II database 1e-24, BlastP 1e-49), a putative bacteriocin immunity protein (BlastP 1e-62, conserved domain pfam08951 5.15e-06), a putative mutacin IV structural protein (BAGEL3 bacteriocin II database 3e-16) and a potential bacteriocin structural protein (BlastP 0.0).

***Streptococcus infantarius* subsp. *infantarius* ATCC BAA 102 –** This genome contains a 14 gene, 10361 bp area of interest containing multiple bacteriocin-related genes (Figure S2). This area of interest included genes predicted to encode a putative bacteriocin secretion accessory protein (BlastP 9e-147, conserved domain TIGR01000 4.66e-92), a putative peptide ABC transporter ATP-binding protein (BlastP 0.0, conserved domain TIGR01193 0.0), a putative infantaricin E precursor (BlastP 1e-47, conserved domain pfam10439 1.74e-03, BAGEL3 bacteriocin II database BlpJ 23-22), a putative infantaricin D precursor (BlastP 4e-33, BAGEL3 bacteriocin II database BlpI 2e-23), a putative infantaricin E precursor (BlastP 4e-08, BAGEL3 bacteriocin II database Thermophilin A 7e-11), a putative CAAX amino terminal protease family protein (BlastP 1e-12, conserved domain pfam02517 1.56e-10), a putative bacteriocin secretion protein (BlastP 6e-09), a putative bovicin 255 peptide precursor (BlastP 2e-33), a putative enterocin A immunity protein (BlastP 1e-61, conserved domain pfam08951 8.78e-06), a putative bacteriocin transporter (BlastP 1e-45), a putative ubericin A structural protein (BlastP 1e-25, BAGEL3 bacteroicin II database 8e-31), a putative bacteriocin immunity protein (BlastP 2e-60, conserved domain pfam08651 1.06e-08), a putative BlpU bacteriocin structural protein (BAGEL3 bacteriocin II database 2e-14, BlastP 2e-08), and a putative class II bacteriocin peptide, possibly bovicin 255 (BAGEL3 bacteriocin II database 1e-10, BlastP 9e-37). Interestingly, this strain has been identified previously as containing genes predicted to be involved in nisin U immunity [[4](#_ENREF_4)].

***Streptococcus* sp. 2_1_36FAA** - This genome contained a possible 5 gene, 2781 bp class IIc bacteriocin cluster (Figure S2). The area of interest contained genes predicted to encode a putative MutR-like transcriptional regulator (BlastP 0.0, conserved domain cd00093 2.04e-06), a putative gassericin A-like structural protein (BlastP 7e-33, conserved domain pfam12173 2.96e-16, BAGEL3 bacteriocin II database 5e-13), a 0070utative membrane protein predicted by BAGEL3 to play a role in bacteriocin modification (BlastP 4e-76), a putative ABC transporter ATP-binding protein (BlastP 0.0, conserved domain cd03230 2.75e-50) and an ABC-2 family transporter protein (BlastP 7e-106, conserved domain pfam13346 1.94e-21) similar to the BviB gene involved in production of Butyrivibriocin AR10 [[5](#_ENREF_5)].

***Lactobacillus acidophilus* ATCC 4796** – This genome contained a helveticin J-like structural gene (BAGEL3 bacteriocin III database 0.0, BlastP 0.0). However, the area of interest (Figure S2) lacked the proposed immunity gene as identified by Joerger and Klaenhammer [[6](#_ENREF_6)].

The genome also contained an enterolysin A-like structural gene (BAGEL3 bacteriocin III database 5e-63, BlastP 2e-157) (Figure S2).

***Lactobacillus antri* DSM 16041** – This genome contained an area of interest (Figure S2) containing two putative Enterolysin A-like genes (BAGEL3 bacteriocin III database 2e-22; 4e-21) containing M23 peptidase domains (conserved domain pfam01551 2.25e-09; 1.90e-11) and a hypothetical protein containing a C39 peptidase family domain predicted to be involved in bacteriocin processing (conserved domain cd02549 1.5e-18). Also present in this cluster are genes predicted to encode a putative glycosyltransferase 1 family protein (BlastP 0.0, conserved domain cd03811 1.72e-49) and a putative glycosyltransferase 2 family protein (BlastP 1e-93, conserved domain cd04196 4.65e-94), neither of which have any apparent bacteriocin-related activity.

***Lactobacillus brevis* subsp *gravensensis* ATCC 27305** – This genome contained a 5 gene, 3691 bp plantaricin cluster (Figure S2). The area of interest contained genes predicted to encode a putative bacteriocin ABC-transporter (BlastP0.0, conserved domain TIGR01193 0.0), a bacteriocin secretion accessory family protein similar to the SppE protein associated with sakacin P (BlastP 1e-08, conserved domain TIGR01000 4.14e-15) [[7](#_ENREF_7)], a putative plantaricin NC8 beta peptide (BlastP 4e-05, BAGEL3 bacteriocin II database 3e-11) and putative a plantaricin NC8 alpha peptide (BlastP e-08, BAGEL3 bacteriocin II database 6e-14). Manual annotation revealed a possible bacteriocin immunity protein (BlastP 6e-09). The area of interest looked quite similar overall to the PLNC8 locus described by Maldonado *et al* [[8](#_ENREF_8)].

***Lactobacillus delbrueckii* subsp *lactis* DSM 20072** - This genome contained an area of interest Figure S2) containing a putative Enterolysin A structural gene (BlastP 0.0, BAGEL3 bacteriocin III database 4e-61).

***Lactobacillus helveticus* DSM 20075** – This genome contained 2 AOIs (Figure S2), each containing a putative helveticin J structural gene (BlastP 1e-168, BAGEL3 bacteriocin III database 1e-172; BlastP 0.0, BAGEL3 bacteriocin III database 5e-177), however neither was accompanied by the hypothesized immunity gene described by Joerger and Klaenhammer [[6](#_ENREF_6)].

***Lactobacillus ultunensis* DSM 16047** – This genome contained 2 putative helveticin J structural genes (BlastP 2e-168, BAGEL3 bacteriocin III database 2e-173; BlastP 0.0, BAGEL3 bacteriocin III database 2e-170). Manual annotation could not identify an immunity gene in either case (Figure S2).

This genome also contained a putative enterolysin A structural gene (BlastP 7e-70, BAGEL3 bacteriocin III database 7e-64) (Figure S2).

**Fusobacteria-assocaited PBGCs**

***Fusobacterium ulcerans* ATCC 49185** – This genome contained a putative Linocin M18 structural gene (BlastP 9e-83) (Figure S1).

***Fusobacterium varium* ATCC 27725** – This genome contained a putative Linocin M18 structural gene (BlastP 4e-80) (Figure S1).

**Synergistetes-associated PBGCs**

***Anaerobaculum hyfrogeniformans* ATCC BAA 1850** – This genome contained a putative Linocin M18 structural gene (BlastP 1e-69, BAGEL3 bacteriocin III database 2e-66) (Figure S1).

***Synergistes* sp. 3_1_syn1** – This genome contained a putative Linocin M18 structural gene (BlastP 2e-66, BAGEL3 bacteriocin III database 2e-56) (Figure S1).

**Proteobacteria-associated PBGCs**

***Arcobacter butzleri* JV22** – This genome contained a putative colicin E9 structural gene (BAGEL3 bacteriocin III database 7e-30, BlastP 2e-38) (Figure S3).

***Klebsiella* sp. MS 92 3** – This genome contained a two gene area of interest covering 1977 bp (Figure S3). BAGEL3 identified the structural gene as a colicin (Bagel3 bacteriocin III database 3e-39) but manual annotation determined it was more likely to be Klebicin B (BlastP 0.0). The second gene in the area of interest was identified as a putative Klebicin B immunity protein (BlastP 1e-66).

***Providencia rettgeri* DSM 1131** – This genome contained a 2 gene cluster predicted to encode a Colicin A structural gene (BAGEL3 bacteriocin III database 1e-42, conserved domain pfam01024 1.23e-41, BlastP 1e-36) and a hypothetical protein predicted by BAGEL3 to be involved in colicin immunity (Figure S3).

***Yokenella regensburgei* ATCC 43003** - This genome contained a 5 gene, 5666 bp microcin gene cluster (Figure S3). The area of interest contained genes predicted to encode an MceJ microcin modification enzyme (BlastP 0.0), a MceI microcin activation protein (BlastP 2e-77, conserved domain pfam02794 8.70e-30), a putative MchE microcin secretion protein (BlastP 0.0, conserved domain pfam13437 9.01e-08), a MchF microcin secretion/processing ATP-binding protein (BlastP 0.0, conserved domain COG2274 0.0), and a putative hemolysin (conserved domain COG3042, 1.06e-23). Unfortunately a microcin structural gene could not be identified.

***Escherichia coli* MS_110_3** – This genome contained 2 potential colicin gene clusters (Figure S3). The first was a 2 gene cluster containing a putative Colicin structural gene (BAGEL3 bacteriocin III database 2e-36, conserved domain pfam12639 2.05e-07) and a putative colicin immunity protein (BlastP 4e-62, conserved domain pfam01320 3.38e-35).

The second was also a 2 gene cluster containing a putative colicin structural gene (BAGEL3 bacteriocin III database 0.0) identified by BlastP as Colicin-Ia (BlastP 0.0, conserved domain pfam01024 7.51e-78) and a putative colicin immunity protein (BlastP 7e-73, conserved domain pfam03526 1.61e-04) (Figure S3).

***Escherichia coli* MS_117_3** - This genome contained a 4 gene, 3568 bp colicin cluster (Figure S3) containing genes predicted to encode a colicin B activity protein (BAGEL3 bacteriocin III database 0.0, BlastP 0.0, conserved domain pfam01024 1.00e-89), a putative colicin B immunity protein (BlastP 4e-127, conserved domain pfam03857 1.14e-54), a putative colicin M activity protein (BAGEL3 bacteriocin III database 0.0, BlastP 0.0, conserved domain pfam14859 1.07e-173) and a putative colicin M immunity protein (BlastP 5e-106, conserved domain pfam13995 1.20e-36).

This genome also contained a 2 gene colicin gene cluster (Figure 3c) containing genes encoding a colicin-Ia structural protein (BAGEL3 bacteriocin III database 0.0, BlastP 0.0, conserved domain 1.29e-77) and a putative colicin-Ia immunity protein (BlastP 2e-73, conserved domain pfam03526 1.56e-04).

***Escherichia coli* MS_119_7** – This genome contained a 4 gene, 2216 bp colicin cluster (Figure S3) containing a colicin B activity protein (BAGEL3 bacteriocin III database 7e-13, BlastP 4e-21, conserved domain pfam01024 8.80e-06), a colicin B immunity protein (BlastP 5e-92, conserved domain pfam03857 1.93e-35), a colicin M activity protein (BAGEL3 bacteriocin III database 0.0, BlastP 0.0, conserved domain pfam14859 1.38e-173) and a colicin M immunity protein (BlastP 5e-106, conserved domain pfam13995 1.20e-36).

***Escherichia coli* MS_124_1** – This genome contained a 2 gene colicin cluster containing a putative Colicin E9 structural gene (BAGEL3 bacteriocin III database 2e-126, BlastP 0.0) and a putative colicin immunity protein (BlastP 2e-53, conserved domain pfam01320 1.80e-49) (Figure S3).

***Escherichia coli* MS_146_1** – This genome contained a putative linocin M18 structural protein (BAGEL3 bacteriocin III database 7e-108, BlastP 0.0) (Figure S3).

***Escherichia coli* MS_153_1** – This genome contained a 6 gene, 3486 bp colicin gene cluster (Figure S3) containing a colicin structural gene (BAGEL3 bacteriocin III database Colicin E9 2e-33, BlastP 0.0, conserved domain cd00085 3.58e-05), a colicin immunity protein (BlastP 1e-63, conserved domain pfam01320 1.51e-32), a colicin structural gene (BlastP 6e-32, conserved domain cd00085 8.91e-07), a colicin immunity protein (BlastP 5e-58, conserved domain pfam01320 1.71e-38), a colicin-like bacteriocin (conserved domain pfam12639 1.61e-07) and a colicin immunity protein (BlastP 2e-62, conserved domain pfam01320 1.32e-36).

***Escherichia coli* MS_16_3** – This genome contained three possible colicin clusters. The first was a 6 gene, 3501 bp colicin cluster (Figure S3) containing, a colicin structural gene (BAGEL3 bacteriocin III database 3e-38, BlastP 3e-172, conserved domain cd00085 2.43e-05), a colicin immunity protein 7e-58, conserved domain pfam01320 3.79e-38), a HNH endonuclease (BlastP 2e-31), a colicin immunity protein (BlastP 2e-62, conserved domain pfam01320 4.52e-36), a HNH endonuclease (BlastP 2e-40, conserved domain pfam12639 2.30-07) and a colicin immunity protein (BlastP 5e-61, conserved domain pfam01320 2.45e-32).

The second was a 2 gene colicin cluster (Figure S3) containing a colicin structural protein (BAGEL3 bacteriocin III database Colicin E9 8e-27, BlastP 5e-24, conserved domain cd00085 2.37e-07), and a colicin E2 immunity protein (BlastP 8e-54, conserved domain pfam01320 6.63e-45).

The third was a 4 gene, 3661 bp colicin cluster (Figure S3) containing a colicin B activity protein (BAGEL3 bacteriocin III database 0.0, BlastP 0.0, conserved domain pfam03515 5.18e-102), a colicin B immunity protein (BlastP 4e-127, conserved domain pfam03857 1.14e-54), a colicin M activity protein (BAGEL3 bacteriocin III database 0.0, BlastP 0.0, conserved domain pfam14859 1.07e-173) and a colicin M immunity protein (BlastP 5e-106, conserved domain pfam13995 1.20e-36).

***Escherichia coli* MS_185_1 ­**– This genome contained a 6 gene, 3544 bp colicin cluster (Figure S3) consisting of a colicin structural gene (BAGEL3 bacteriocin III database Colicin E9 2e-33, BlastP 0.0, conserved domain cd00085 3.61e-05), a colicin immunity protein (BlastP 1e-63, conserved domain pfam01320 1.51e-32), a colicin protein (BlastP 2e-31, conserved domain cd00085 1.07e-06), a colicin immunity protein (BlastP 5e-58, conserved domain pfam01320 1.71e-38), a HNH endonuclease (BlastP 8e-36, conserved domain pfam12639 1.74e-07) and a colicin immunity protein (BlastP 2e-62, conserved domain pfam01320 1.32e-36).

***Escherichia coli* MS_196_1** – This genome contained a 2 gene colicin gene cluster (Figure S3) containing a colicin structural gene (BAGEL3 bacteriocin III database Colicin-10 3e-84, BlastP 0.0, conserved domain pfam01024) and a colicin immunity protein (BlastP 1e-70, conserved domain pfam03526 2.55e-15).

***Escherichia coli* MS_200_1** – This genome contained a 6 gene, 3583 bp colicin gene cluster (Figure S3) containing a colicin protein (BAGEL3 bacteriocin III database 2e-36, BlastP 0.0, conserved domain cd00085 3.05e-05), a colicin immunity protein (BlastP 7e-58, conserved domain pfam01320 3.79e-38), a HNH endonuclease domain protein (BlastP 2e-43, conserved domain pfam12639 4.82e-07), a colicin immunity protein (BlastP 2e-50, conserved domain pfam01320 1.86e-26), a HNH endonuclease domain protein (BlastP 2e-41, conserved domain pfam12639 2.30e-07) and a colicin immunity protein (BlastP 5e-61, conserved domain pfam01320 2.45e-32).

***Escherichia coli* MS_21_1** – This genome contained a 4 gene, 2767 bp colicin cluster (Figure S3) containing a colicin structural gene (BAGEL3 bacteriocin III database 5e-24, BlastP 0.0, conserved domain pfam06958), a colicin immunity protein (BlastP 7e-60, conserved domain pfam01320 8.77e-39), a colicin structural gene (BAGEL3 bacteriocin III database 3e-09) and a colicin immunity protein (BlastP 1e-60, conserved domain pfam01320 1.39e-31).

***Escherichia coli* MS_45_1** – This genome contained a 6 gene, 3454 bp colicin cluster (Figure S3) containing a colicin E9 protein (BAGEL3 bacteriocin III database 2e-33, BlastP 0.0, conserved domain cd00085 3.58e-05), a colicin immunity protein (BlastP 1e-63, conserved domain pfam01320 1.51e-32), a HNH endonuclease domain protein (BlastP 1e-62, conserved domain cd00085), a colicin immunity protein (BlastP 5e-58, conserved domain pfam01320 1.71e-38), a HNH endonuclease (BlastP 8e-44, conserved domain pfam12639 1.74e-07) and a colicin immunity protein (BlastP 2e-62, conserved domain pfam01320 1.32e-36).

This genome also contained a 6 gene, 6641 bp microcin gene cluster in this strain (Figure S3). The area of interest includes genes predicted to encode a Microcin H47 structural gene (BAGEL3 bacteriocin I database 6e-43, BlastP 1e-56), a MchC microcin biosynthesis family protein (BlastP 0.0), a putative microcin H47 activating protein (BlastP 4e-95, conserved domain pfam02794 6.05e-36), a microcin H47 secretion protein mchE (BlastP 0.0, conserved domain pfam13437 3.91e-11), ABC transporter protein MchF (BlastP 0.0, conserved domain COG2274 0.0), and a microcin M structural gene (BAGEL3 bacteriocin I database 4e-61, BlastP 3e-54).

***Escherichia coli* MS_57_2** – This genome contained a 6 gene, 3462 bp colicin gene cluster (Figure S3) including genes predicted to encode a colcin E9 structural gene (BAGEL3 bacteriocin III database 2e-35, BlastP 0.0, conserved domain cd00085 8.361e-06), a colicin immunity protein (BlastP 5e-66, conserved domain pfam01320 7.31e-33), a HNH endonuclease protein (5e-62, conserved domain cd00085 1.11e-06), a colicin immunity protein (BlastP 5e-58, conserved domain pfam01320 1.71e-38), a HNH endonuclease (BlastP 3e-43, conserved domain pfam12639 1.74e-07) and a colicin immunity protein (BlastP6e-62, conserved domain pdam01320 3.02e-37).

***Escherichia coli* MS_78_1** – This genome contained a 3 gene, 2462 bp colicin gene cluster (Figure S3) containing genes predicted to encode a colicin structural protein (BAGEL3 bacteriocin III database 3e-124, BlastP 0.0, conserved domain cd00085 1.77e-05), a colicin immunity protein (BlastP 1e-53, conserved domain pfam01320 1.80e-49) and a colicin lysis protein (BlastP 9e-31, conserved domain pfam02402 8.05e-13).

***Escherichia coli* MS_85_1** – This genome contained a 2 gene colicin cluster (Figure S3) containing genes predicted to encode a colicin structural protein (BAGEL3 bacteriocin III database 2e-126, BlastP 0.0, conserved domain cd00085 1.50e-5) and colicin immunity protein (BlastP 1e-53, conserved domain pdam01320 1.80e-49).

***Escherichia coli* SE11** - This genome contained a 2 gene colicin cluster (Figure S3) containing genes predicted to encode a colicin structural protein (BAGEL3 bacteriocin III database 0.0, BlastP 0.0, conserved domain pfam01024 3.73e-76) and a colicin immunity protein (BlastP 2e-73, conserved domain pfam03526 1.56e-04).

***Escherichia* sp. 3_2_53FAA** - This genome contained a 6 gene, 3464 bp colicin cluster (Figure S3) containing genes predicted to encode a colicin structural protein (BAGEL3 bacteriocin III database 2e-36, BlastP 0.0, conserved domain cd00085 3.59e-06), a colicin immunity protein (BlastP 7e-60, conserved domain pfam01320 8.77e-39), a colicin protein/HNH endonuclease (BAGEL3 bacteriocin III database 1e-12, BlastP 2e-43, conserved domain pfam12639 2.05e-07), a colicin immunity protein (BlastP 4e-62, conserved domain pfam01320 3.38e-35), a colicin protein/HNH endonuclease (BAGEL3 bacteriocin III database 5e-09, BlastP 4e-41, conserved domain pfam12639 .30e-07) and a colicin immunity protein (BlastP 5e-61, conserved domain pfam01320 2.45e-32).

**References**

1. Valdes-Stauber N, Scherer S. Isolation and characterization of Linocin M18, a bacteriocin produced by Brevibacterium linens. Appl Environ Microbiol. 1994;60(10):3809-14.

2. McClerren AL, Cooper LE, Quan C, Thomas PM, Kelleher NL, van der Donk WA. Discovery and in vitro biosynthesis of haloduracin, a two-component lantibiotic. Proceedings of the National Academy of Sciences of the United States of America. 2006;103(46):17243-8. doi:10.1073/pnas.0606088103.

3. Clayton EM, Daly KM, Guinane CM, Hill C, Cotter PD, Ross PR. Atypical Listeria innocua strains possess an intact LIPI-3. BMC microbiology. 2014;14:58. doi:10.1186/1471-2180-14-58.

4. Draper LA, Tagg JR, Hill C, Cotter PD, Ross RP. The spiFEG locus in Streptococcus infantarius subsp. infantarius BAA-102 confers protection against nisin U. Antimicrob Agents Chemother. 2012;56(1):573-8. doi:10.1128/aac.05778-11.

5. Kalmokoff ML, Cyr TD, Hefford MA, Whitford MF, Teather RM. Butyrivibriocin AR10, a new cyclic bacteriocin produced by the ruminal anaerobe Butyrivibrio fibrisolvens AR10: characterization of the gene and peptide. Canadian journal of microbiology. 2003;49(12):763-73. doi:10.1139/w03-101.

6. Joerger MC, Klaenhammer TR. Cloning, expression, and nucleotide sequence of the Lactobacillus helveticus 481 gene encoding the bacteriocin helveticin J. Journal of bacteriology. 1990;172(11):6339-47.

7. Urso R, Rantsiou K, Cantoni C, Comi G, Cocolin L. Sequencing and expression analysis of the sakacin P bacteriocin produced by a Lactobacillus sakei strain isolated from naturally fermented sausages. Applied microbiology and biotechnology. 2006;71(4):480-5. doi:10.1007/s00253-005-0172-x.

8. Maldonado A, Ruiz-Barba JL, Jimenez-Diaz R. Purification and genetic characterization of plantaricin NC8, a novel coculture-inducible two-peptide bacteriocin from Lactobacillus plantarum NC8. Appl Environ Microbiol. 2003;69(1):383-9.
