# Supplementary material for: In silico identification of bacteriocin gene clusters in the gastrointestinal tract, based on the Human Microbiome Project’s reference genome database
Source: BMC Microbiol. 2015 Sep 16;15:183. doi: 10.1186/s12866-015-0515-4 (PMC4573289; doi:10.1186/s12866-015-0515-4)
Supplement: Supplementary file 3 [file 12866_2015_515_MOESM3_ESM.docx]

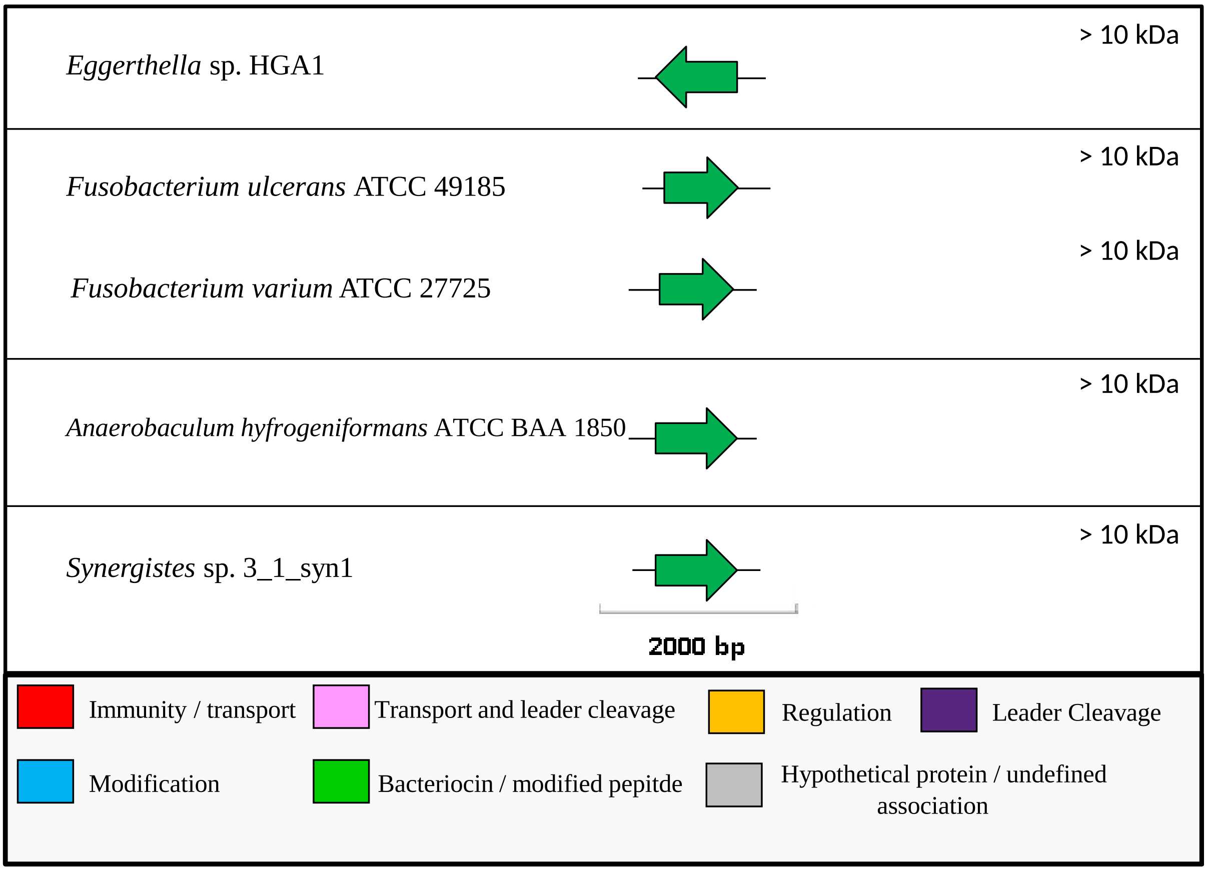

Figure S1. Diagrammatic representation of remaining PBGCs identified in the Actinobacteria, Fusobacteria and Synergistetes.
