# Supplementary material for: In silico identification of bacteriocin gene clusters in the gastrointestinal tract, based on the Human Microbiome Project’s reference genome database
Source: BMC Microbiol. 2015 Sep 16;15:183. doi: 10.1186/s12866-015-0515-4 (PMC4573289; doi:10.1186/s12866-015-0515-4)
Supplement: Supplementary file 4 [file 12866_2015_515_MOESM4_ESM.docx]

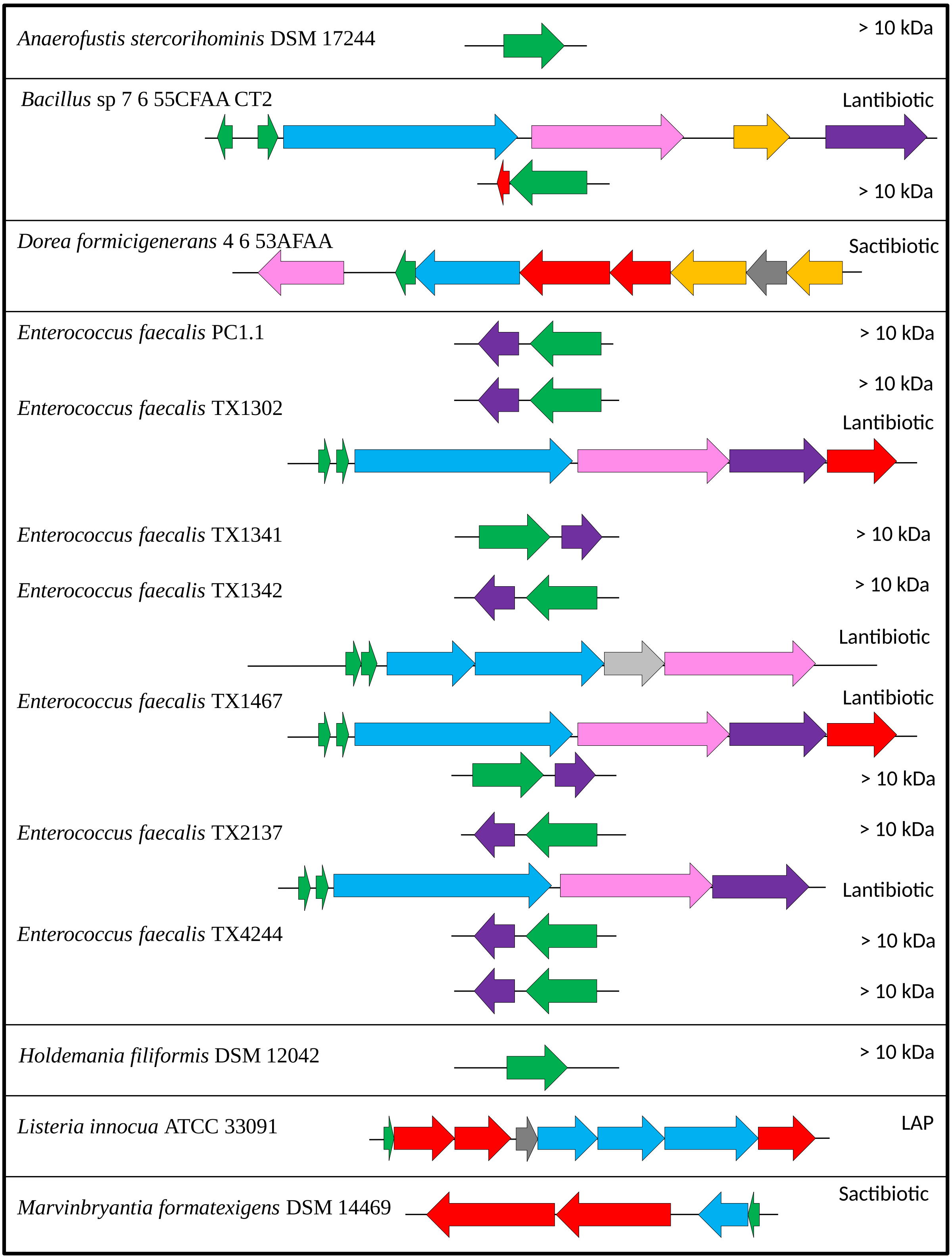
Anaerofustis stercorihominis DSM 17244
> 10 kDa
Bacillus sp 7 6 55CFAA CT2
Lantibiotic
> 10 kDa
Dorea formicigenerans 4 6 53AFAA
Sactibiotic
Enterococcus faecalis PC1.1
> 10 kDa
Enterococcus faecalis TX1302
> 10 kDa
Lantibiotic
Enterococcus faecalis TX1341
> 10 kDa
Enterococcus faecalis TX1342
> 10 kDa
Lantibiotic
Enterococcus faecalis TX1467
Lantibiotic
> 10 kDa
Enterococcus faecalis TX2137
> 10 kDa
Lantibiotic
Enterococcus faecalis TX4244
> 10 kDa
> 10 kDa
Holdemania filiformis DSM 12042
> 10 kDa
Listeria innocua ATCC 33091
LAP
Marvinbryantia formatexigens DSM 14469
Sactibiotic

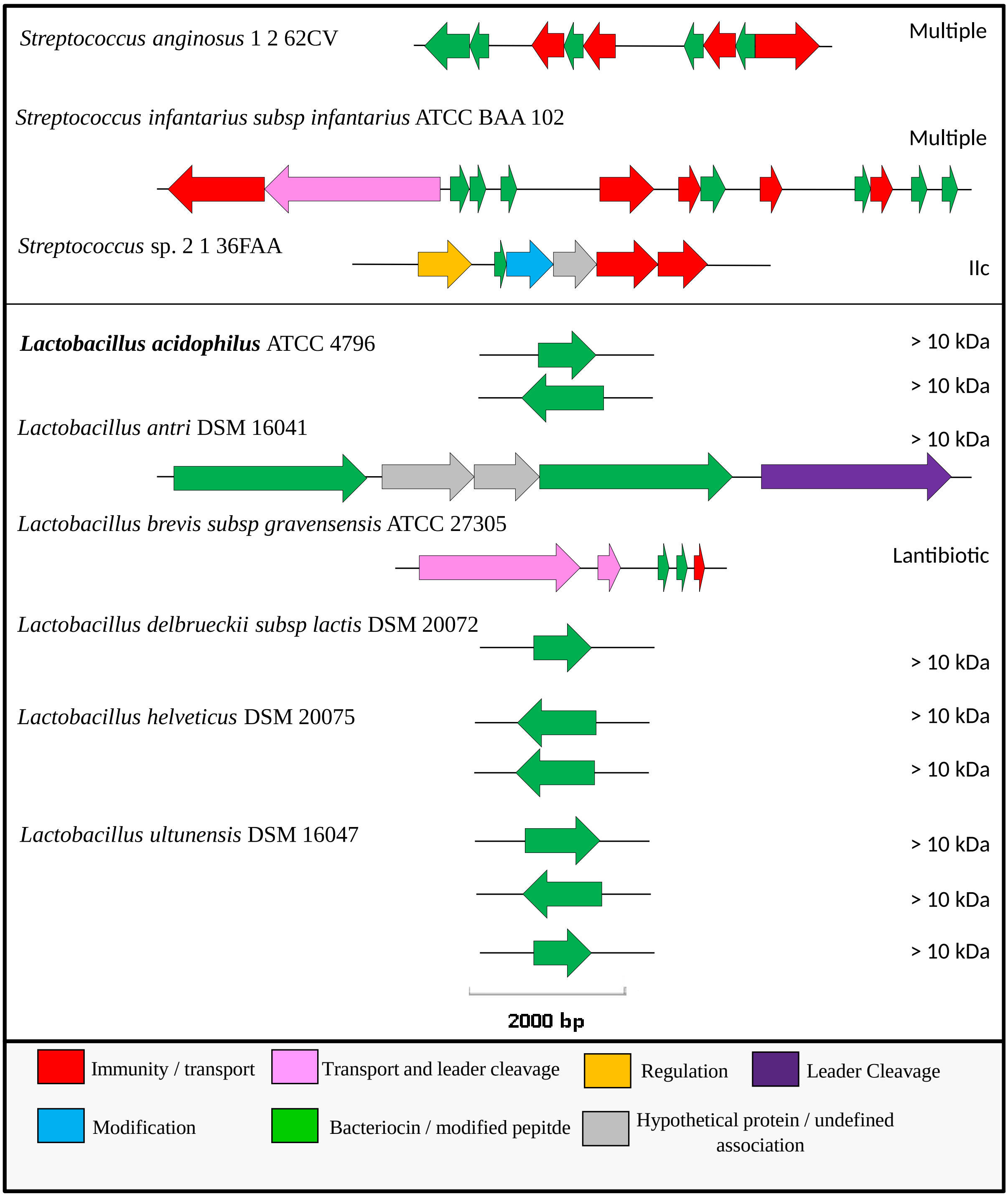

Figure S2. Diagrammatic representation of remaining PBGCs identified in the Firmicutes.
