# Supplementary material for: In silico identification of bacteriocin gene clusters in the gastrointestinal tract, based on the Human Microbiome Project’s reference genome database
Source: BMC Microbiol. 2015 Sep 16;15:183. doi: 10.1186/s12866-015-0515-4 (PMC4573289; doi:10.1186/s12866-015-0515-4)
Supplement: Supplementary file 5 [file 12866_2015_515_MOESM5_ESM.docx]

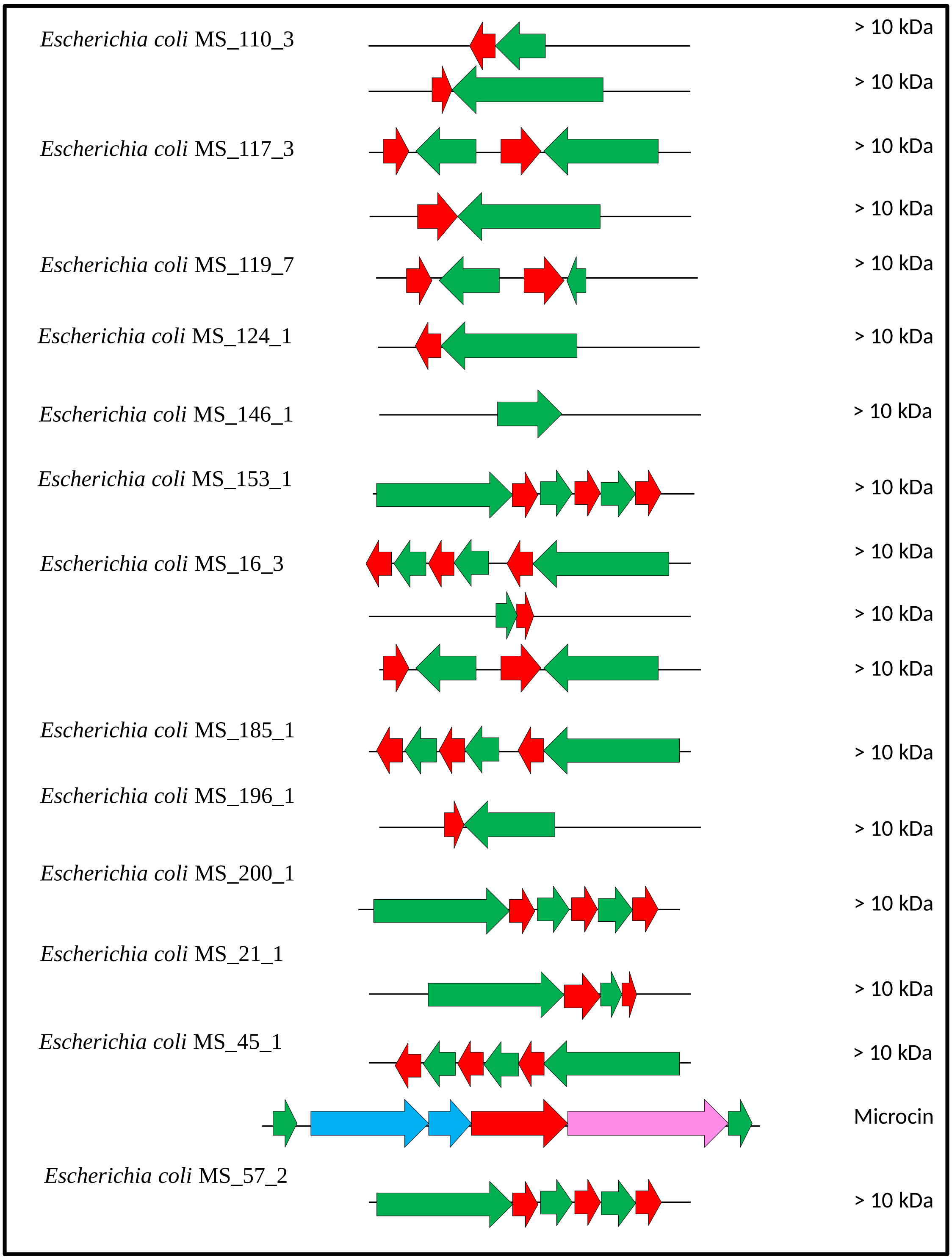
Escherichia coli MS_110_3
> 10 kDa
> 10 kDa
Escherichia coli MS_117_3
> 10 kDa
> 10 kDa
Escherichia coli MS_119_7
> 10 kDa
Escherichia coli MS_124_1
> 10 kDa
Escherichia coli MS_146_1
> 10 kDa
Escherichia coli MS_153_1
> 10 kDa
Escherichia coli MS_16_3
> 10 kDa
> 10 kDa
> 10 kDa
Escherichia coli MS_185_1
> 10 kDa
Escherichia coli MS_196_1
> 10 kDa
Escherichia coli MS_200_1
> 10 kDa
Escherichia coli MS_21_1
> 10 kDa
Escherichia coli MS_45_1
> 10 kDa
Microcin
Escherichia coli MS_57_2
> 10 kDa

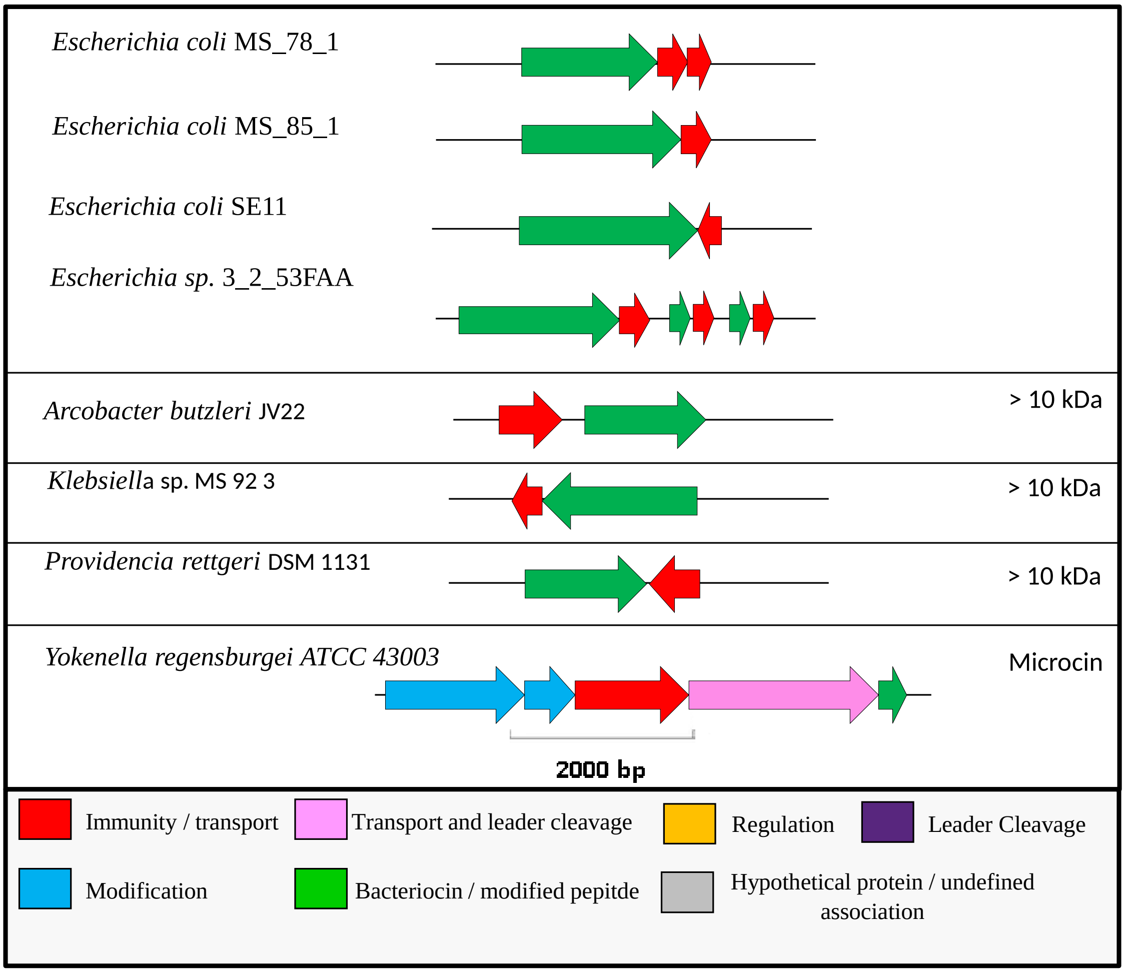

Figure S3. Diagrammatic representation of remaining PBGCs identified in the Proteobacteria.
